# Acute sleep deprivation disrupts emotion, cognition, inflammation, and cortisol in young healthy adults

**DOI:** 10.3389/fnbeh.2022.945661

**Published:** 2022-09-23

**Authors:** Kayla I. Thompson, Minh Chau, Margaret S. Lorenzetti, Lauren D. Hill, Ana I. Fins, Jaime L. Tartar

**Affiliations:** ^1^Department of Psychology and Neuroscience, Nova Southeastern University, Davie, FL, United States; ^2^Department of Clinical and School Psychology, Nova Southeastern University, Davie, FL, United States; ^3^Icahn School of Medicine at Mount Sinai, New York City, NY, United States; ^4^Department of Psychology, Florida International University, Miami, FL, United States

**Keywords:** sleep deprivation, inflammation, emotion, cognition, cortisol, neurobehavioral functioning

## Abstract

Chronic sleep deprivation has been demonstrated to diminish cognitive performance, alter mood states, and concomitantly dysregulate inflammation and stress hormones. At present, however, there is little understanding of how an acute sleep deprivation may collectively affect these factors and alter functioning. The present study aimed to determine the extent to which 24-h of sleep deprivation influences inflammatory cytokines, stress hormones, cognitive processing across domains, and emotion states. To that end, 23 participants (mean age = 20.78 years, SD = 2.87) filled out clinical health questionnaires measured by the Pittsburgh Sleep Quality Index, Morningness Eveningness Questionnaire, and Center for Epidemiological Studies Depression Scale. Actigraph was worn for seven days across testing to record sleep duration. At each session participants underwent a series of measures, including saliva and blood samples for quantification of leptin, ghrelin, IL-1β, IL-6, CRP, and cortisol levels, they completed a cognitive battery using an iPad, and an emotion battery. We found that an acute sleep deprivation, limited to a 24 h period, increases negative emotion states such as anxiety, fatigue, confusion, and depression. In conjunction, sleep deprivation results in increased inflammation and decreased cortisol levels in the morning, that are accompanied by deficits in vigilance and impulsivity. Combined, these results suggest that individuals who undergo 24 h sleep deprivation will induce systemic alterations to inflammation and endocrine functioning, while concomitantly increasing negative emotions.

## Introduction

Sleep serves a critical role to human health and is commonly viewed as a restorative process. Previous work has emphasized sleep's involvement in maintaining immune functioning, metabolic conservation, psychological well being, and cognitive functioning. Despite the important role good sleep hygiene plays in health and well being, the deleterious effects of sleep loss are commonly overlooked. Unfortunately, poor sleep health has become a normalized experience and characteristic feature of modern society. Sleep loss is characterized as insufficient sleep time, often ≤ 7 h of sleep per 24-h period (Altevogt and Colten, 2006). There is growing evidence to suggest that sleep disturbance and deprivation is a health concern and threat to society in the United States (Schoenborn and Adams, 2010). This is particularly prevalent among medical professionals, shift workers, flight personnel, graduate students, not to mention older adults who have sleep disturbances that go untreated. Many professional settings, however, require adequate functioning to execute complex tasks which involve but are not limited to attention, executive functioning, and the ability to regulate emotion. Studies using human and non-human animal samples have established that acute and chronic sleep deprivation leads to deleterious changes in neurobehavioral functioning, such as induced anxiety states and deficits in attention and memory, warranting further understanding of the systemic consequences sleep deprivation ensues (Tononi and Cirelli, 2006; Chee and Chuah, 2007; Kahn-Greene et al., 2007; Yang et al., 2014; Mishra et al., 2016; Kaur et al., 2017; Manchanda et al., 2018; Tomaso et al., 2021).

It has been shown that sleep deprivation can influence neurobehavioral outcomes through altering the inflammatory response and neuroendocrine stress system (Haack et al., 2007; Irwin et al., 2008; Vgontzas et al., 2013), thereby inducing symptoms such as anxiety and aggravating cognitive performance (Chee and Chuah, 2007; Kahn-Greene et al., 2007; Kahn et al., 2013). With respect to inflammation, pro-inflammatory cytokines are typically investigated, and findings suggests widespread immune changes following poor sleep. For instance, in an animal study using male Wistar rats, chronic sleep deprivation induced elevated inflammatory levels of Tumor Necrosis Factor alpha (TNF- α) and interleukin (IL)-1β, which led to anxiety-like behavior and cognitive deficits (Manchanda et al., 2018). Similarly, another study using a rat model indicated that acute sleep deprivation was linked to a global decrease of the following pro-inflammatory markers: IL-6, TNF- α, IL-1β, and Monocyte Chemoattractant Protein-1 (MCP-1) (Bajaj and Kaur, 2022). Humans comparably exhibit alterations in inflammatory markers under poor sleep conditions. There is a trend for elevations in inflammatory markers that is more readily apparent in humans relative to non-human animals. Following acute sleep deprivation in a human sample of healthy subjects, studies have shown a significant increase in proinflammatory markers such as IL-6 and IL-1β (Frey et al., 2007; Haack et al., 2007; Vgontzas et al., 2007; Sauvet et al., 2010; Abedelmalek et al., 2013). Of note, IL-6 is implicated in acute immune responses and the secretion of C-reactive protein (CRP), which also has pro-inflammatory activity and are both frequently altered following sleep deprivation.

As a testament to the consistent findings implicating IL-6 and CRP changes after sleep deprivation, a systematic review exploring the relationship between sleep deprivation and inflammatory markers in humans, solely focused on these two markers. The study yielded similar results found in non-human animal studies following chronic sleep deprivation which showed an increase in IL-6 and CRP (Irwin et al., 2016). Interestingly, these findings on acute sleep deprivation showed no effects on inflammation markers; accordingly, the author suggested that acute sleep deprivation may not influence the inflammatory signaling pathway. Nevertheless, other studies have shown elevated inflammation markers following an acute sleep deprivation (Kato et al., 2000; Shearer et al., 2001; Meier-Ewert et al., 2004; Dimitrov et al., 2006; Irwin et al., 2006, 2008; Bajaj and Kaur, 2022). Compared to IL-6, IL-1β has remained an understudied variable, with respect to sleep deprivation, despite evidence suggesting an intimate relationship between the two. In fact, Jewett and Krueger (2012) asserted that IL-1β promotes non-rapid eye movement (NREM) sleep, and therefore can induce sleepiness and fatigue, alongside decreased cognition, in both humans and non-human animals. Hence, studies have found altered levels of IL-1β in the face of sleep deprivation. In the absence of sleep deprivation, IL-1β, like IL-6, follows a diurnal pattern with lower levels throughout the day and peak levels at night. Given this, in an experimental study, one night of sleep deprivation resulted in the absence of the IL-1β nocturnal rise (Covelli et al., 1992), conversely a more recent study by Tartar et al. (2015) demonstrated elevated levels in a chronic sleep restricted group. In agreement with these results were the findings by Frey et al. (2007), suggesting that 40 h of an acute sleep deprivation induced a significant increase in IL-1β. Nevertheless, it is worth noting, there are still inconsistent results within the literature. For example, studies such as Sauvet et al. (2010) found low levels of IL-1 β after sleep deprivation. Changes in IL-1β with sleep loss is commonly studied in rat models which generally find IL-1β elevations with chronic sleep deprivation and decreased levels after total sleep deprivation (Manchanda et al., 2018; Bajaj and Kaur, 2022). Combined, elevated levels in inflammation markers would plausibly account for mood changes observed following acute sleep deprivation (Benson et al., 2017), as neuroimaging supports the finding that peripheral inflammation contributes to behavioral changes (Felger, 2018). Furthermore, the inflammatory pathway has been implicated in influencing cognitive functioning in healthy young adults and most notably, older adults. For example, CRP and IL-6 were shown to associate with reduced cognitive performance (Frydecka et al., 2015; Tegeler et al., 2016; Vintimilla et al., 2019). These changes in immune functioning in turn, are likely related to a dysregulation in cortisol release that occurs with sleep loss (Spiegel et al., 1999).

Regarding neuroendocrine functioning, rats in the aforementioned acute sleep deprivation condition exhibited a reduction in cortisol levels and an increase in both leptin and insulin (Bajaj and Kaur, 2022). With that said, pro-inflammatory markers are known to have endocrine and metabolic effects (Agorastos et al., 2014). Once the stress system is activated, inflammatory cytokines and cortisol levels are altered, creating a chain effect once one system experiences dysregulation (Yeager et al., 2011; Jones and Gwenin, 2021). Cortisol is a primary stress biomarker that is controlled by the hypothalamic–pituitary adrenal (HPA) axis. Along with changes in inflammation, sleep and circadian rhythmicity are crucial in the regulation of the HPA axis (Guyon et al., 2014). Accordingly, sleep deprivation has been shown to have an effect on cortisol levels as the end product of HPA axis activity (Vgontzas et al., 2004; Omisade et al., 2010; Thorsley et al., 2012; Song et al., 2015; Wright et al., 2015). Whereas, experimental evidence in humans indicates that cortisol levels elevate in response to acute sleep deprivation (Balbo et al., 2010; Omisade et al., 2010), a range of studies have found that cortisol levels decrease (Weibel et al., 1995; Gronfier et al., 1997, 1998; Leproult et al., 1997; Spiegel et al., 1999, 2004a; Omisade et al., 2010; Guyon et al., 2014), which is more consistent with controlled findings in animal models (Bajaj and Kaur, 2022). To that point, endocrine hormones, ghrelin and leptin are also implicated in the stress response ensued by sleep deprivation and have regulatory effects on the HPA axis secretion of cortisol (Omisade et al., 2010). A systematic review of studies in humans concluded that leptin levels decrease following an acute stressor such as sleep deprivation (Bouillon-Minois et al., 2021), while ghrelin levels increase (Spiegel et al., 2004b; Bali and Singh Jaggi, 2016). Ostensibly, the combination of these changes explains the weight gain that occurs with poor sleep hygiene, as leptin is a hormone released from an adipocyte tissue that signals satiety (i.e., an appetite suppressing hormone) and ghrelin signals hunger to the brain (i.e., an appetite stimulating hormone). Although studies have supported the notion that sleep has an effect on neuroendocrine functioning, the results are ambiguous in the direction of these effects on cortisol, thereby limiting our general understanding of the effect on the neuroendocrine system. In other words, the research has been limited to examining one variable of the system, rather than an integrative analysis.

Despite the consequences of sleep deprivation being well-documented, the overall effects are not well-understood under varying sleep conditions, namely an acute sleep deprivation. Taken together, between non-human animal and human studies, there is widespread inconsistency regarding the impact of endocrine and immune functioning on biological markers, including the well-studied markers, IL-6 and cortisol. Furthermore, as it stands, IL-1β remains understudied in the context of acute sleep deprivation, despite evidence showing its possible implication. One of the complications in understanding the effects of poor sleep health is that the HPA axis, appetite system, and immune system are part of a complex extended endocrine-immune network where each system can modify one another. As such, there is considerable inconsistency in the literature regarding acute sleep deprivation. Consequently, additional research is needed to understand the systemic effects on health and well being. The current study examined the effects of 24 h of sleep deprivation on markers of inflammation, stress hormones, cognition, and emotion in healthy young adults with no prior history of sleep disturbance. Our goal was to provide a comprehensive multi-methodological approach that goes beyond a single marker approach and explain how disruption of the inflammatory and hormonal pathway has neurobehavioral effects. This provides the opportunity to see how the isolated effect of one night of an acute deprivation can affect multiple systems.

## Materials and methods

### Participants

This study was carried out according to a protocol approved by the Nova Southeastern University (NSU) Institutional Review Board. Twenty-three participants were recruited (*n* = 23; 9 females, 14 males, μ age=20.78, SD =2.87), of which all read and signed a written informed consent. Following consent, all participants completed the Epworth Sleepiness Scale (ESS), Pittsburgh Sleep Quality Test (PSQI), Morningness Eveningness Questionnaire (MEQ), and Center for Epidemiological Studies Depression Scale (CESD) as a pre-screening tool. Exclusionary criteria included scores indicative of sleep disorder, sleep disturbance, or depression. The cutoff scores were as follows: ESS ≥10 (Johns, 1991), PSQI >7(Carpenter and Andrykowski, 1998; Beck et al., 2004), CESD ≥16 (Lewinsohn et al., 1997). Mental health status was assessed using a pre-screening questionnaire for history and current diagnosis. No participants were excluded based on these criterions. Instructions were verbally provided to refrain from caffeine intake at least 24 h before testing. Compensation of $100 Visa gift card was provided for participant's time.

### Procedure

#### Sleep monitoring and sleep deprivation

**Figure 1:**
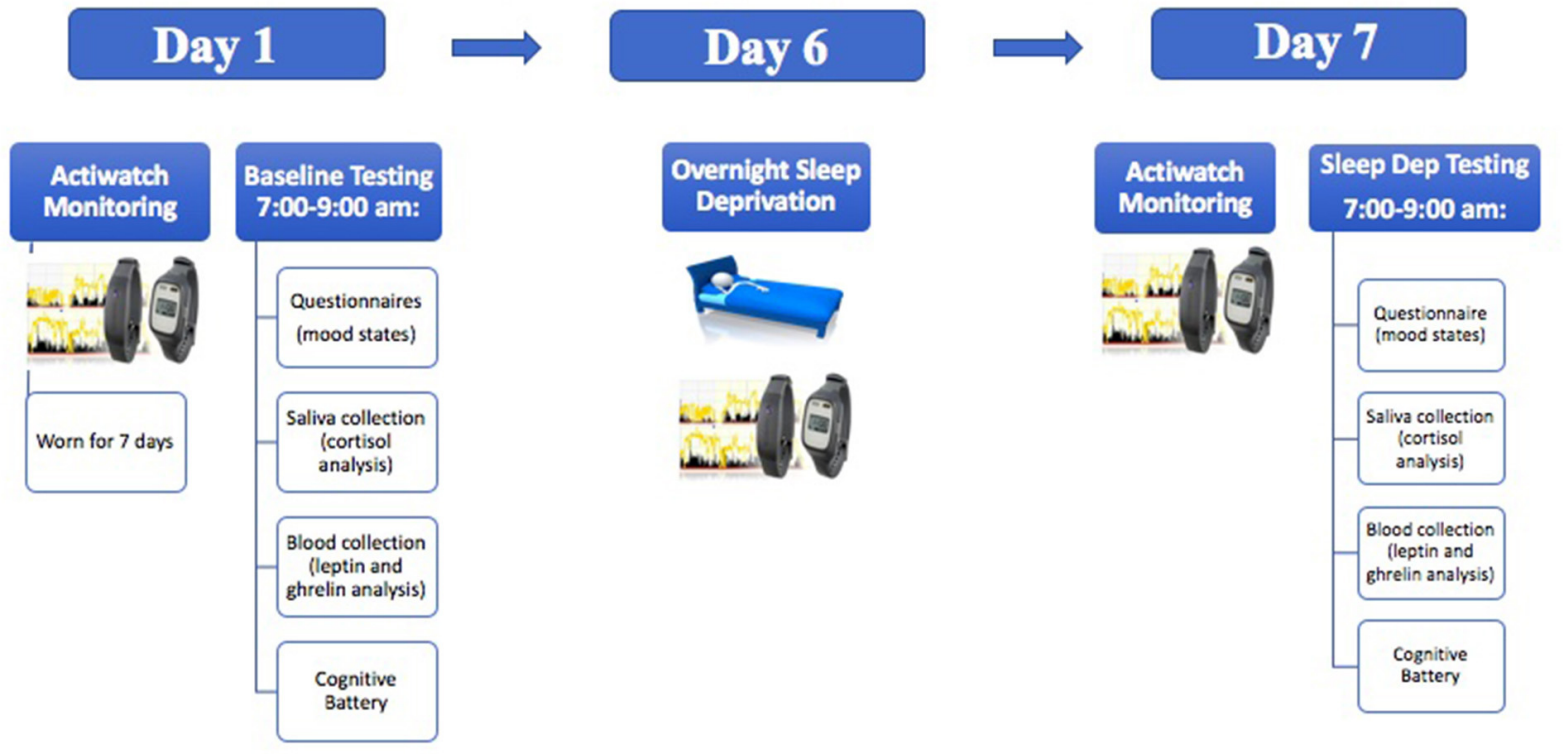
A schematic illustration demonstrating the experimental timeline.

Testing occurred between 7:00 and 9:00 a.m. and included one baseline testing session and one sleep deprivation testing session seven days later (see Figure 1). Total sleep time was calculated through the use of Actiwatch wrist monitors and Actiware software (Phillips Respironics, New Jersey). Actiwatch data were also used to verify that participants were awake during the day of sleep deprivation before they arrived to the laboratory. During the sleep deprivation session, the participants came to the laboratory at 9:00 p.m. for overnight total sleep deprivation. The participants were constantly monitored by 2–4 researchers throughout the evening. In addition, all participants wore actigraphy monitors throughout the sleep deprivation period. During sleep deprivation, the participants were permitted to engage in non-stressful activities (e.g., talking, board games etc.,). Only water was permitted during the sleep deprivation period (no other beverages or food was permitted).

At each testing session, the participants provided saliva samples for cortisol quantification. Saliva was also collected from each participant by unstimulated passive drool for salivary cortisol analysis (participants drooled directly into a 1.5 mL microcentrifuge tube through a small sterile cylinder). Following this, 3 mLs of blood was taken through venipuncture. Blood was collected into EDTA coated tubes. Immediately after collection, the tubes were centrifuged for 10 min at 1,500 x g at 4°C. The plasma was then apportioned into 0.5 ml aliquots and stored at −20°C until analyses were conducted.

#### Biomarker quantification

##### Cortisol, CRP, Il-6, and Il-1β

Saliva tubes were placed in a freezer following participant testing and stored at −20°C. Saliva samples were run in duplicate and quantified *via* human enzyme immunoassay kits per the manufacturer's instructions (Salimetrics LLC, USA: Cat# 1-3102, RRID:AB_2801306). The samples were immediately read in a BioTek ELx800 plate reader (BioTek Instruments, Inc, USA) at 450 nm with a correction at 630 nm. All samples were within the detection ranges indicated in the immunoassay kits. The variation of sample readings was within the expected limits. Final concentrations for the biomarkers were generated by interpolation from the standard curve in μg/dL for cortisol and pg/mL for CRP, IL-6, and IL-1β.

##### Leptin and ghrelin

Aliquoted plasma samples were placed in a freezer following participant testing and stored at −20°C. Saliva samples were run in duplicate and quantified *via* human enzyme immunoassay kits per the manufacturer's instructions for leptin (R&D systems, Inc. USA, Cat# DLP00, RRID:AB_2783014) and ghrelin (Thermo Fisher Scientific Inc., USA, Cat# BMS2192, RRID:AB_2575470). The samples were immediately read in a BioTek ELx800 plate reader (BioTek Instruments, Inc, USA) at 450 nm. All samples were within the detection ranges indicated in the immunoassay kits. The variation of sample readings was within the expected limits. Final concentrations for the biomarkers were generated by interpolation from the standard curve in pg/mL for ghrelin and ng/mL for leptin.

#### Cognitive measures

Cognition testing was conducted using the automated “Cognition” test battery from Joggle Research (Joggle Research, Seattle WA). The Joggle Cognition battery consists of eight cognitive measures administered on a standard electronic tablet (Apple IPad). Total testing time is ~20 min, which prevents participant fatigue. The cognition test battery consists of eight tasks covering a diverse set of cognitive domains (e.g., executive function, episodic memory, complex cognition, and sensorimotor speed) and are based on tests known to activate specific brain systems (Basner et al., 2015). The tests include a Psychomotor Vigilance Test (PVT), the Balloon Analog Risk Task (BART). the Digital Symbol Substitution Task (DSST), the Line Orientation Task (LOT), an Abstract Matching (AM) test, the number back (NBACK) task, a Visual Object Learning Task (VOLT), a Motor Praxis Task (MPT).

#### Emotion measures

##### State-Trait anxiety inventory (STAI-Y)

State and Trait anxiety were measured using the STAI-Y (Spielberger et al., 1983). Each scale is composed of 20 questions that tap stable aspects of an individual's general pre-disposition to experience anxiety symptoms and 20 items that focus on transitory emotional/anxious arousal. Items are rated on a four-point Likert scale. The instrument shows adequate reliability and validity (Spielberger et al., 1983).

##### Profile of Mood States (POMS)

The POMS is a psychometrically sound instrument that measures acute mood (“How do you feel right now”) and ongoing mood (“How have you been feeling during the past week, including today”) (McNair et al., 1971). The measure consists of 65 adjectives rated by participants on a five-point likert scale that asked participants about their mood in the past week. The 65 items yield six subscales: anger–hostility, confusion–bewilderment, depression–dejection, fatigue–inertia, tension–anxiety, and vigour–activity. A Total Mood Disturbance (TMD) score is also calculated based on the scores of each subscale. The range for each scale is as follows: Anger (0–48), Confusion (0–28), Depression (0–60), Fatigue (0–28), Tension (0–36), Vigour (0–32), and TMD (−32–100).

### Baseline clinical health questionnaires

Depression, sleep quality, and chronotype were assessed using the Center for Epidemiologic Studies Depression Scale (CES-D), Pittsburgh Sleep Quality Index (PSQI), and the Morningness–Eveningness Questionnaire (MEQ), respectively. The CES-D is a short self-report measure shown to be reliable and valid across a variety of demographic characteristics in the general population (Radloff, 1977). This measure consists of 20 items asking questions about the frequency of symptoms associated with depression in the past week, with items rated on a four-point Likert scale. A score of 16 or greater is indicative of possible depression. Sleep quality was assessed using the Pittsburgh Sleep Quality Index (PSQI) (Buysse et al., 1989), a self-report instrument comprised of 19 items evaluating seven components of sleep over the past month: subjective sleep quality, sleep latency, sleep duration, habitual sleep efficiency, sleep disturbances, daytime dysfunction, and use of sleep medications. The seven components can be summed to yield a global score that ranges from 0 to 21. Generally, higher scores indicate poorer sleep quality, and a global score >5 is suggestive of poor sleep quality. The instrument exhibits adequate psychometric properties (Buysse et al., 1989). The Morningness–Eveningness Questionnaire (MEQ) is a widely administered self-report measure composed of 19 items used to determine if one's peak sleepiness and alertness is in the morning or in the evening (Horne and Östberg, 1976).

### Statistical analyses

The effect of sleep deprivation on each of our continuous variables were individually analyzed using paired samples *t*-tests at baseline and post for participants' performance of neurocognitive measures, mood states, stress hormones, and inflammation. All reported *p*-values are two-tailed with an a priori significance level of *p* < 0.05. Effect size are reported using Cohen's d and interpreted small (*d* = 0.2), medium (*d* = 0.5), and large (*d* = 0.8) according to the recommendations set forth by Cohen (1988). Before conducting the statistical analyses, preliminary checks on statistical assumptions were verified. Using the Schapiro-Wilk test, the assumption of normal distribution was met for most, but not all variables, warranting the inclusion of non-parametric, Wilcoxon signed ranked tests in these instances. All data were analyzed using SPSS statistical package version 25 (IBM, NY, USA, RRID:SCR_016479).

### Follow up correlation analyses

Given that baseline average sleep duration was moderately lower than expected (see results), this prompted us to carry out a follow-up analysis on self-report and actigraphy sleep measures in order determine if the low average sleep duration (prior to SD) had any bearing on our outcome measures. To that end, we conducted a correlation analysis to estimate the relation between total sleep time and all outcome measures. Correlation analyses were also conducted using the subjective measure of the PSQI and all outcome measures. Due to the number of correlations calculated, a Bonferroni correction was implemented. One-way ANOVAs were also conducted to determine the effect of MEQ (morning, intermediate, and evening chronotypes) on all outcome measures.

## Results

### Actigraphy

Although participants were instructed to sleep 8 h each night prior to sleep deprivation, sleep behavior was still objectively verified the week prior to sleep deprivation through actigraphy monitoring to ensure that the participants were not experiencing sleep loss the week prior to experimental sleep deprivation. Actigraphy recording showed that the total sleep time was only 6 h and 51 min (SD = 1.24), falling slightly below the 8 h instruction. Although, the total sleep time was in line with typical habitual sleep (Belenky et al., 2003; Rupp et al., 2009; Broussard et al., 2015) for this age group's patterns. Participants averaged an awake time of 7:32 a.m., with times ranging from 4:58 to 7:45 a.m.

### Biomarkers of inflammation and hormonal function

**Figure 2:**
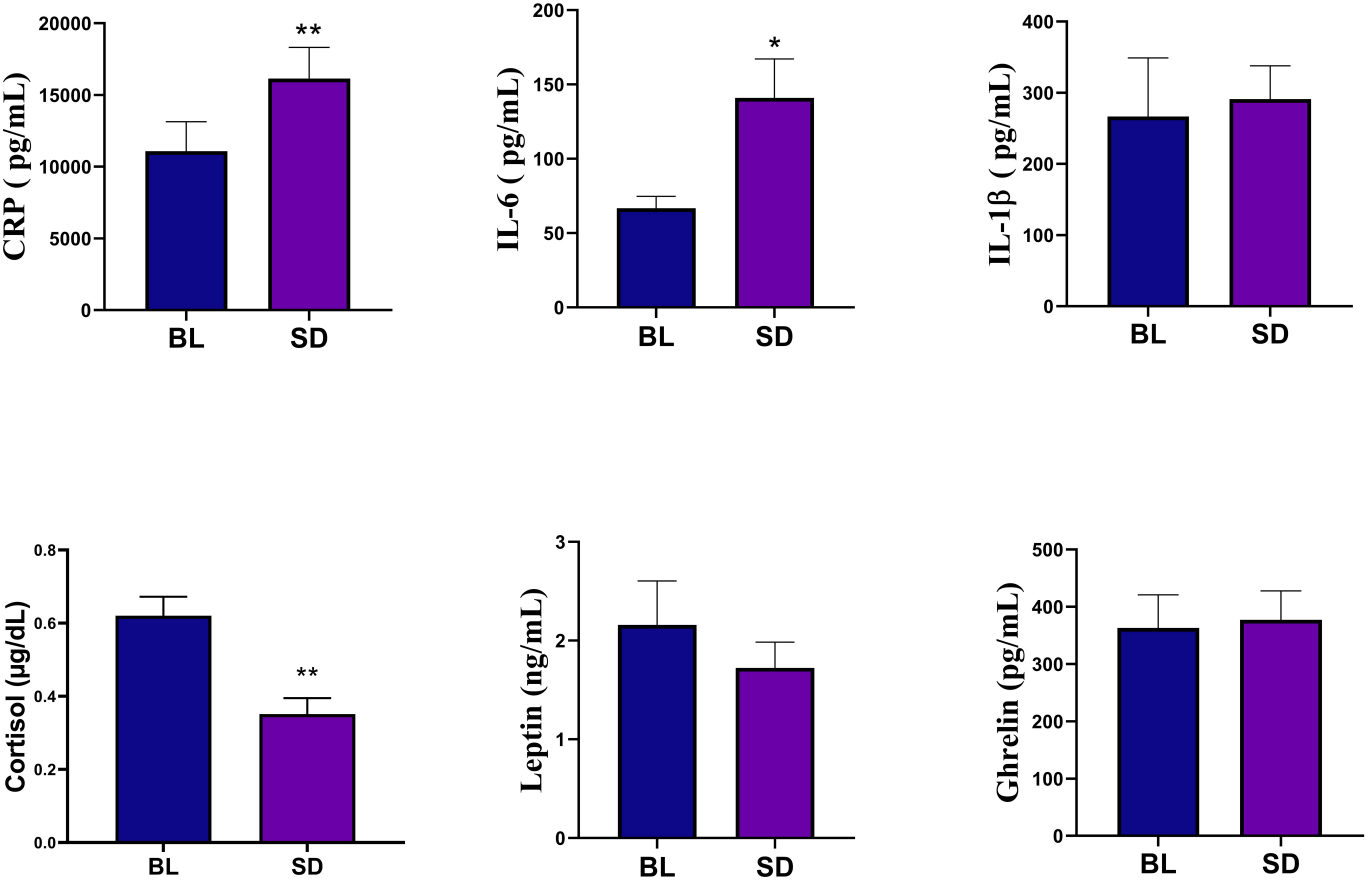
Biomarker analyses showed that compared to baseline (BL), the sleep deprivation (SD) condition significantly increased in C-Reactive Protein (CRP) and IL-6. There was also a significant decrease in cortisol levels. Errors bars represent SEM and asterisks indicate *p* < 0.01.

Paired samples *t*-tests revealed that relative to baseline (mean = 66.78, SD = 37.86), IL-6 levels were significantly increased following one night of sleep deprivation (mean = 140.95, SD = 125.48), *t*_(22)_ = −3.031, *p* < 0.01, *d* = 0.63. Following sleep deprivation, CRP levels (mean = 16148.84, SD = 10423.49) were also significantly increased relative to baseline (mean = 11080.38, SD = 9848.60), *t*_(22)_ = −3.412, *p* < 0.01, *d* = 0.71. There was no effect of sleep deprivation on IL-1β, *t*_(22)_ = 0.414, *p* = 0.683, although IL-1β levels at baseline (mean = 266.51, SD = 395.41) were relatively lower to the night of deprivation (mean = 290.89, SD = 225.49). Significant differences emerged when examining morning cortisol levels at baseline *versus* post-sleep deprivation showing a reduction, some of which has been previously reported in Trivedi et al. (2017), *t*_(22)_ = 5.196, *p* < 0.01, *d* = 1.083. There were no significant changes in leptin relative to baseline *t*_(22)_ = 1.149, *p* = 0.263, nor were changes observed in ghrelin *t*_(22)_ = −0.362, *p* = 0.721. Figure 2 shows the means and SEs for the biomarkers. Assumptions of normality were violated when examining leptin and IL-1β, however non-parametric tests yielded results consistent with the paired samples *t*-test, leptin *p* = 0.054, and IL-1β *p* = 0.346.

### Cognitive functioning

Table 1 shows means and standard deviations for cognition measures.

**Table 1:** Cognitive functioning.

		**Baseline Mean ±SD**	**Sleep deprivation Mean ±SD**	***T*-value**	***P*-value**	**Cohen's d**
**PVT**	RT	285.98 ± 27.79	316.09 ± 74.54	−2.142	*p* =0.044	0.447
	Lapses	2.83 ± 2.55	2.74 ± 3.29	1.28	*p* = 0.214	0.024
	FS	2.69 ± 2.63	2.65 ± 3.31	0.058	*p* = 0.954	0.012
**BART**	RT	737.76 ± 900.03	367.19 ± 327.08	2.868	*p* = 0.009	0.598
	Total pumped	136.65 ± 19.64	133.69 ± 27.70	0.640	*p* ≥ 0.05	0.113
	Total ballpop	12.73 ± 2.13	12.21 ± 3.074	0.972	*p* > 0.05	0.203
**DSST**	RT	925.23 ± 115.21	951.99 ± 140.13	−1.44	*p* > 0.05	0.301
	CR	86.04 ± 9.71	84.47 ± 12.33	0.983	*p* > 0.05	0.205
**LOT**	RT	7169.69 ± 3099.28	6300.22 ± 3340	2.36	*p* = 0.028	0.491
	CR	12.43 ± 3.96	11.91 ± 4.15	0.619	*p* = 0.542	
	EC	3.41 ± 1.86	2.94 ± 2.39	0.090	*p* = 0.180	0.289
**VOLT**	RT	2184.93 ± 824.35	1785.52 ± 500.06	3.005	*p* = 0.007	0.627
	CR	16.08 ± 1.59	16.73 ± 1.76	−1.845	*p* = 0.079	0.385
**MPT**	RT	494.79 ± 78.96	459.82 ± 52.51	2.37	*p* = 0.027	0.494
**NBACK**	RT	615.88 ± 91.22	603.53 ± 87.38	0.471	*p* > 0.05	0.098
	CR	47.83 ± 8.25	49.91 ± 6.71	−1.44	*p* >0.05	0.302
**AM**	RT	2125.97 ± 1006.95	1683.74 ± 501.38	2.85	*p* = 0.009	0.594
	CR	16.96 ± 2.74	17.21 ± 3.32	−0.371	*p* = 0.714	0.077

This table demonstrates the means, standard deviations, p-values, and effects sizes for pre- and post-tests on cognitive measures using the computerized Joggle tests.

RT, Reaction time; CR, Correct responses; FS, False starts; Total pumped, Total ballons pumped; Total ballpop, Total balloons popped; and EC, excess clicks.

#### Psychomotor Vigilance Task (PVT)

Upon conducting a paired sample *t*-tests to examine performance post-sleep deprivation (mean = 316.09, SD = 74.54), it was revealed that the mean reaction time were significantly increased relative to baseline (mean = 285.98, SD = 27.79), *t*_(22)_ = −2.142, *p* = 0.044, *d* = 0.447. There were no significant differences in lapses [*t*_(22)_ = 1.28, *p* = 0.214] and false starts [*t*_(22)_ = 0.058, *p* = 0.954]. Assumptions of normality were violated when examining mean reaction time and false start, however non-parametric tests yielded results consistent with the paired samples *t*-test, mean reaction time *p* = 0.024, and false start *p* = 0.774.

#### Balloon Analog Risk Task (BART)

Upon conducting a paired sample *t*-tests to examine performance on the Balloon Analog Risk Task (BART) post-sleep deprivation (mean = 367.19, SD = 327.08), it was revealed that the mean reaction time were significantly decreased relative to baseline (mean = 737.76, SD = 900.03), *t*_(22)_ = 2.868, *p* = 0.009, *d* = 0.598. There were no significant differences in total balloons pumped and popped. Assumptions of normality were violated when examining mean reaction time, however non-parametric tests yielded results consistent with the paired samples *t*-test, mean reaction time *p* < 0.001.

#### Digital Symbol Substitution Task (DSST)

Performance on the Digital Symbol Substitution Task was examined post-sleep deprivation using a paired samples *t*-test. Results yielded no significant difference in number of correct responses or reaction time. Assumptions of normality were violated when examining correct responses, however non-parametric tests yielded results consistent with the paired samples *t*-test, correct responses *p* = 0.425.

#### Line Orientation Task

Performance on the Line Orientation Task (LOT) was examined post-sleep deprivation using a paired samples *t*-test. Results revealed that mean reaction time was significantly decreased following one night of sleep deprivation (mean = 6300.22, SD = 3340) relative to baseline (mean = 7169.69, SD = 3099.28), *t*_(22)_ = 2.36, *p* = 0.028, *d* = 0.491. There were no significant changes in number of correct responses, *t*_(22)_ = 0.619, *p* = 0.542 and mean excess clicks, *t*_(22)_ = 0.090, *p* = 0.180. Assumptions of normality were violated when examining mean reaction time, however non-parametric tests yielded results consistent with the paired samples *t*-test, mean reaction time *p* = 0.008.

#### Visual Object Learning Task

Upon conducting a paired sample *t*-tests to examine performance on the Visual Objet Learning Task (VOLT) post-sleep deprivation (mean = 1785.52.19, SD = 500.06), it was revealed that the mean reaction time were significantly decreased relative to baseline (mean = 2184.93, SD = 824.35), *t*_(22)_ = 3.005, *p* = 0.007, *d* = 0.627. There was no significant difference in the number of correct responses *t*_(22)_ = −1.845, *p* = 0.079. Assumptions of normality were violated when examining mean reaction time, however non-parametric tests yielded results consistent with the paired samples *t*-test, mean reaction time *p* = 0.003.

#### Motor Praxis Task

Performance on the Motor Praxis Task (MPT) was examined post-sleep deprivation using a paired samples *t*-test. Results yielded a significant reduction in mean reaction time *t*_(22)_ = 2.37, *p* = 0.027, *d* = 0.494.

#### NBACK

There were no significant differences on the NBack (all *p*'s > 0.05).

#### Abstract Matching (AM)

Upon conducting a paired sample *t*-tests to examine performance on Abstract Matching (AM) post-sleep deprivation (mean = 1683.74, SD = 501.38), it was revealed that the mean reaction time were significantly decreased relative to baseline (mean = 2125.97, SD = 1006.95), *t*_(22)_ = 2.85, *p* = 0.009, *d* = 0.594. There was no significant difference in the number of correct responses *t*_(22)_ = −0.371, *p* = 0.714.

### Emotion measures

**Figure 3:**
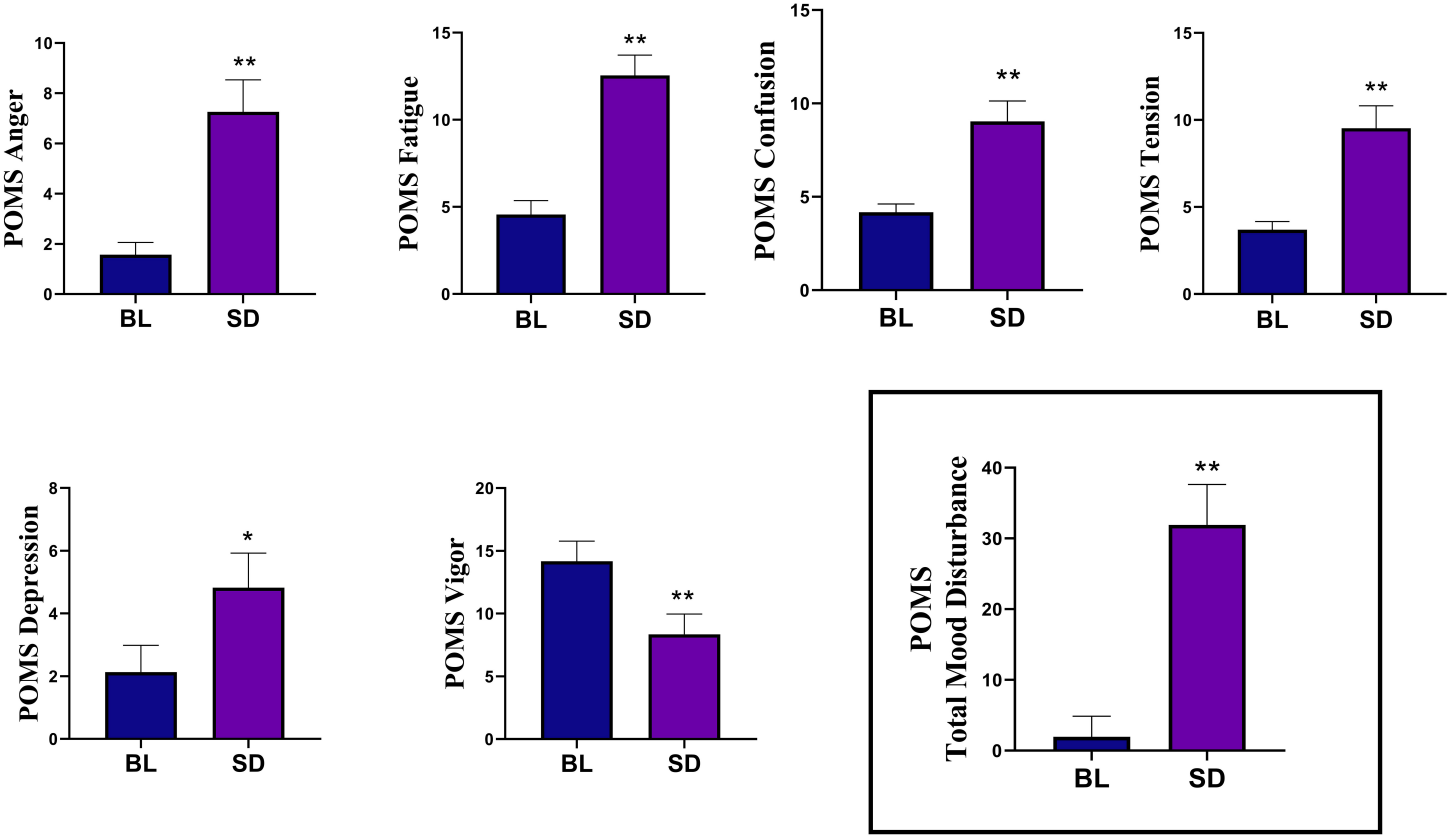
The results of the Profile of Mood States (POMS) measures showed that compared to baseline (BL), sleep deprivation (SD) significantly increased in tension, depression, anger, fatigue, and confusion. There was a significant decrease in vigor. The Total Mood Disturbance composite score was also significantly increased. Errors bars represent SEM, asterisks indicates *p* < 0.05, and double asterisks indicate *p* < 0.01.

**Figure 4:**
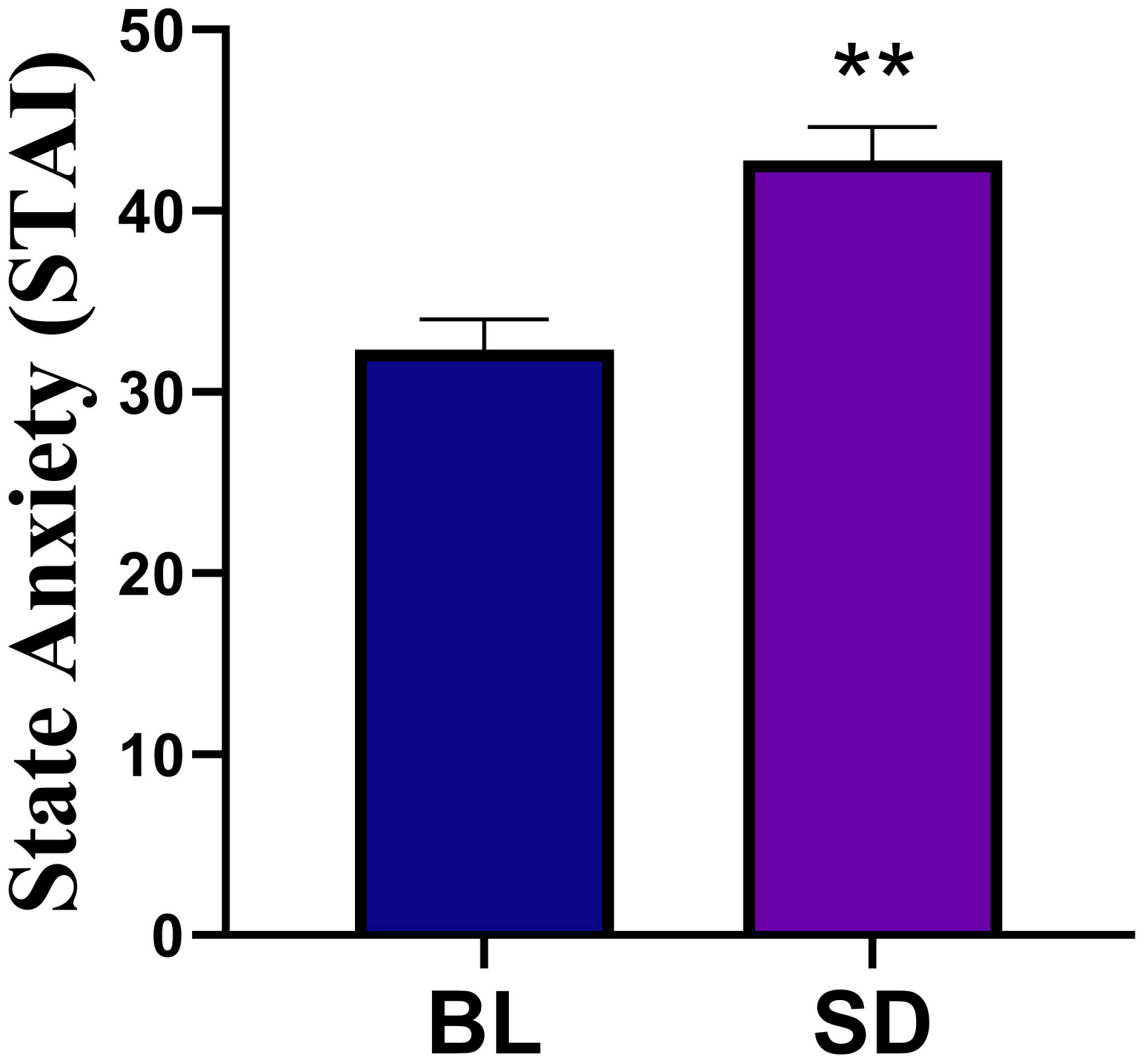
Compared to baseline (BL) there was a significant increase in state anxiety following sleep deprivation. Errors bars represent SEM and double asterisks indicate *p* < 0.01.

The results of the Profile of Mood States (POMS) data are shown in Figure 3. POMS measures showed that compared to baseline, there was a significant increase in tension [*t*_(22)_ = −4.09, *p* < 0.001, *d* = 0.854], depression [*t*_(22)_=-2.355, *p* = 0.028, *d* = 0.491], anger [*t*_(22)_ = −3.99, *p* < 0.001, *d* = 0.831], fatigue [*t*_(21)_ = −5.86, *p* < 0.001, *d* = 1.25], confusion [*t*_(22)_ = −4.24, *p* < 0.001, *d* = 0.89], and TMD [*t*_(20)_ = −5.49, *p* < 0.001, *d* = 1.20]. There was a significant decrease in vigor, [*t*_(22)_ = 4.81, *p* < 0.001, *d* = 0.99]. Compared to baseline (mean = 32.35, SD = 7.91), there was a significant increase in state anxiety (see Figure 4) following sleep deprivation (mean = 42.78, SD = 8.90), *t*_(22)_ = −5.012, *p* < 0.001, *d* = 1.045.

#### Associations between sleep and outcome measures

Pearson correlation analysis revealed no significant correlation between self-reported or actigraphy-measured sleep and the outcome measures. This indicates that prior sleep behavior, whether normal (TST 7–9 h) or dysfunctional, did not have any bearing on the biochemical measures taken at baseline or after sleep deprivation. A one-way ANOVA revealed no effect of MEQ (morning, intermediate, and evening chronotypes) on outcome measures.

## Discussion

The current findings demonstrate that one night of acute sleep deprivation altered circulating markers of systemic inflammation, cortisol, emotion, and cognitive performance. Specifically, we identified a significant decrease in cortisol levels, accompanied by an increase in inflammatory markers, CRP and IL-6, which is consistent with prior findings suggesting sleep modulates immune and endocrine functioning (Leproult et al., 1997; Spiegel et al., 1999, 2004a; Omisade et al., 2010; Guyon et al., 2014; Minkel et al., 2014; Wright et al., 2015; Atrooz and Salim, 2020). Although there were no significant changes in either ghrelin or leptin, there was a trend for leptin to decrease following sleep deprivation, while ghrelin trended towards an increase. In general, changes to leptin and ghrelin are related to increased metabolic demands of sleep deprivation. We also found an increase in negative mood ratings and impulsivity, whereas vigilance and sensorimotor speed were decreased. We did not find any effects of sleep deprivation on executive functioning, spatial learning/memory, spatial orientation, abstraction, complex scanning, or concept formation.

While cognitive deficits have been well-documented as a consequence of sleep deprivation (Kahn et al., 2013; Short and Louca, 2015), the present results support the argument that there is a threshold of sleep loss that needs to be reached before higher order cognitive domains are affected (e.g., executive functioning). This also explains why executive functioning is typically impaired with chronic sleep loss but not always with acute sleep loss (Binks et al., 1999; Quigley et al., 2000; Sagaspe et al., 2003, 2006; Drummond et al., 2006; Tucker et al., 2010). Nevertheless, there are mixed findings on the effects of acute sleep deprivation on cognitive performance (Nilsson et al., 2005; Lim and Dinges, 2010; Killgore and Weber, 2014; Chua et al., 2017; Kusztor et al., 2019), and specific cognitive domains are still disputed. An alternative explanation for the cognitive results is that any decreases induced by sleep deprivation may have been masked by practice effects. Often reaction time on computerized neurocognitive tasks have shown to be increased on post measures, due to familiarity with the task and learning effects (Calamia et al., 2012). Practice effects would elucidate the observable patterns of reduced reaction time across higher order cognitive tasks in our study, such as LOT, AM, NBACK, and VOLT, while not being prone to increased error. Seen through this light, impulsivity, vigilance, and sensorimotor speed may be less susceptible to practice effects under acute sleep deprivation. Hence, studies have suggested that practice effects are minimally related to cognitive domains such as attention (Duff et al., 2012) and that tests within cognitive domains may be less or more resistant to practice (Bartels et al., 2010).

Despite studies suggesting that acute sleep deprivation is not sufficient to initiate inflammatory signaling that can be translated into increased systemic inflammation (Irwin et al., 2016), studies such as ours and others have yielded contrasting results (Kato et al., 2000; Shearer et al., 2001; Meier-Ewert et al., 2004; Dimitrov et al., 2006; Irwin et al., 2006, 2008). Of note, the effect size for CRP was moderately strong and slightly better than those found for Il-6, as seen in Figure 2. These results may suggest that an acute sleep deprivation of one night can induce an increase in toll-like receptor (TLR)−4 production of inflammatory cytokines (Irwin et al., 2006), through activation of the control pathway in the inflammatory signaling cascade, nuclear factor kappa B (NF-κB) (Irwin et al., 2008). It is worth noting, activation of NF-κB leads to subsequent upregulation of inflammatory response genes, as well as the master circadian clock regulator which has an interrelated regulatory network with the HPA axis, modulating glucocorticoid release (Kalsbeek et al., 2006; Oster et al., 2006).

To this end, the reduced cortisol levels following acute sleep deprivation reflect the altered state of the HPA axis, as the morning cortisol peak was not apparent. Few studies have shown similar outcomes in dampened or reduced morning cortisol awakening response (Leproult et al., 1997; Spiegel et al., 1999, 2004a; Omisade et al., 2010; Guyon et al., 2014). Interestingly, symptoms of anxiety associate with blunted cortisol levels in healthy adults across age and sex (Brooks and Robles, 2009; de Rooij et al., 2010; Crişan et al., 2016). This supports the idea that disrupted HPA regulation in response to an acute stress can contribute to altered behavioral and mental health outcomes (Kinlein et al., 2015; Fiksdal et al., 2019). HPA axis dysregulation presented a state effect in response to the physiological stress of sleep deprivation, as evidenced by an increase on the STAI. Considering we did not evaluate cortisol at different time points following deprivation, we were unable to determine if the circadian modulation would have resulted in an elevation in cortisol during the evening as seen in other studies as a demonstration of HPA axis recovery (Leproult et al., 1997; Spiegel et al., 2004a; Omisade et al., 2010). Nevertheless, our findings are critical in showing that sleep deprivation decreases the HPA axis activity. This can result in dysregulation of the circadian rhythm in the peripheral CLOCK through the subsequent release of glucocorticoids. Herein, the HPA pathway is forced to reconfigure its responsiveness under stress due to a possible increase in the negative feedback regulation (Redwine et al., 2000). The effects on peripheral CLOCKS are known to influence the expression of clock related genes which regulates emotions and inflammatory reactions such as the Per2. In both interactions between the CLOCK system and inflammation, as well as the HPA axis, physiologic concentrations of glucocorticoids are necessary for adequate functioning. Yet, one night of sleep deprivation, although not persistent, can induce stress that disrupts the circadian fluctuation produced by the CLOCK system, thereby altering many systemic factors.

The deleterious effects of sleep deprivation lend themselves to widespread altered immune functioning, along with dysregulated cortisol levels which can impact mood (Benson et al., 2017; Bollen et al., 2017; Felger, 2018) and cognition (Frydecka et al., 2015; Tegeler et al., 2016; Vintimilla et al., 2019). Decreased cortisol awakening response (CAR) has been related to the vulnerability of depression (Kuehner et al., 2007). In agreement, the current study found increased depressive symptomatology as well as decreased morning cortisol levels after sleep deprivation. Specifically, participants demonstrated negative mood changes in vigor, tension, depression, anxiety, anger, confusion, and fatigue, which parallels previous findings. Endeavors to overcome the negative effects induced by sleep deprivation during neurocognitive testing becomes arduous, like engaging in daily life activities when sleep deprived. Among the cognitive domains reported to have deficits from sleep deprivation, vigilant attention remains the most prominent which was corroborated in our study by the PVT (Dinges et al., 1997; Doran et al., 2001; Sagaspe et al., 2003; Lim and Dinges, 2008) signifying significantly slower speed. In line with these results, were slower sensorimotor speed in MPT performance. Yet interestingly, lapses and false starts on the PVT were similar between baseline and post-sleep deprivation. In agreement with previous work (Saksvik-Lehouillier et al., 2020), we also found reduced reaction time on the BART which reflects deficits in impulsivity after sleep deprivation. Although, there was no difference on the total balloons pumped or popped suggesting risk decision making remained intact, which contradicts the literature. Reduced reaction time was observed across all other cognitive measures (except PVT). As previously mentioned, these results may have altered due to practice effects or reflective of impulsivity as it has been previously observed following sleep deprivation, due to a speed-accuracy tradeoff (Saksvik-Lehouillier et al., 2020).

The current study provides a unique multi-methodological approach to investigating a 24-h acute sleep deprivation and presents an integrative systems perspective. Nevertheless, there are limitations to this study that are worth mentioning as they provide uncertainty to the results. Caffeine has pro- and anti-inflammatory effects, as well as effects on cognition and mood. Therefore, studies on sleep deprivation should instruct subjects to avoid caffeine. However, despite our initial instructions, there were no objective data collected to ensure participants adhered to this request. Furthermore, caffeine withdrawal could alter the results for subjects that are accustomed to their daily caffeine intake. Caffeine withdrawal symptoms can appear very early after stopped use and last for 2–9 days (Juliano and Griffiths, 2004). These symptoms include, but are not limited to, fatigue, decreased energy, decreased alertness, depressed mood, difficulty concentrating, and irritability. The emotion measures utilized also pose a limitation in identifying whether changes in negative affect are clinically meaningful. Currently, the literature on the POMS lacks anchor-based approaches to identify clinically relevant changes on the scales; therefore, mean improvement scores are generally relied upon (Dworkin et al., 2008). With respect to cortisol measurement, participants individual morning peak were not accounted for when collecting samples, which stems caution in interpreting these results. Future studies would benefit by tailoring the collection time to the individuals usual wake time, to ensure obtaining the morning cortisol peak. Lastly, given the small sample size and expected trends seen in leptin and ghrelin, it is plausible that the study did not yield sufficient power to show these effects. On the otherhand, IL-1β may require sustained levels of deprivation as results did not reveal any pattern. To further derive at a consensus in the literature, it is recommended that these findings are validated in a larger cohort. Moreover, the current study should motivate further investigation into the effects of sleep deprivation using incremental variances of sleep deprivation, ranging from 24 to 48 h., to determine if there are critical limits within sleep deprivation with marked deficits. Future studies may also use incremental measures of cortisol following sleep deprivation to better understand the potential HPA axis recovery period following sleep deprivation.

Combined, these findings advance the understanding of the deleterious effects of an acute sleep deprivation by demonstrating system-wide changes in humans. Given the association between these systemic alterations and age-related pathology, these findings are particularly relevant for understanding the potential health costs of those in careers that commonly involve sleep deprivation, as well as those with untreated or undetected sleep disturbances. To combat these issues, treatments are able to target sleep behaviors which may modify outcomes such as inflammation and improve overall health (Irwin et al., 2014).

## Data availability statement

The raw data supporting the conclusions of this article will be made available by the authors, without undue reservation.

## Ethics statement

The studies involving human participants were reviewed and approved by Nova Southeastern University Institutional Review Board. The patients/participants provided their written informed consent to participate in this study.

## Author contributions

JT and AF designed the study. JT, ML, and LH carried out experimental procedures and participant testing. MC and KT carried out data analyses. KT drafted the manuscript. All authors approved the final version of the manuscript.

## Funding

This study was funded through a Nova Southeastern University President's Faculty Research and Development Grant (#33582) awarded to JT.

## Conflict of interest

The authors declare that the research was conducted in the absence of any commercial or financial relationships that could be construed as a potential conflict of interest.

## Publisher's note

All claims expressed in this article are solely those of the authors and do not necessarily represent those of their affiliated organizations, or those of the publisher, the editors and the reviewers. Any product that may be evaluated in this article, or claim that may be made by its manufacturer, is not guaranteed or endorsed by the publisher.
